# Emerging Infections: What Have We Learned from SARS?

**DOI:** 10.3201/eid1007.040166

**Published:** 2004-07

**Authors:** Alison P. Galvani

**Affiliations:** * University of California, Berkeley, California, USA

**Keywords:** control, emerging diseases, influenza, outbreak, SARS

Given the current size and mobility of the human population, emerging diseases pose a continuing threat to global health. This threat became reality with the outbreak of severe acute respiratory syndrome (SARS). The emergence of a disease requires two steps: introduction into the human population and perpetuated transmission. Although preventing the introduction of a new disease is ideal, containing a zoonosis is a necessity. The lessons that we have learned from SARS were the topic of a meeting of The Royal Society on January 13, 2004, in London, England.

Zoonoses are responsible for most emerging infectious diseases, including infections caused by Ebola virus, West Nile virus, monkeypox, hantavirus, HIV, and new subtypes of influenza A. In the case of SARS coronavirus (SARS-CoV), serologic evidence indicates that the virus was spread through interspecies transmission from wild game markets in Guangdong, China (Malik Peiris, University of Hong Kong). This finding led to bans in the wild meat trade from Nan Shan Zhong (Guangzhou Respiratory Disease Research Institute) similar to the ban on eating nervous system tissue from cows that was implemented after new variant Creutzfeldt-Jakob disease emerged in Britain.

Ecologic changes, concomitant with increasing contact between humans and animal disease reservoirs, contribute to zoonoses. The emergence of SARS was facilitated by increased contact between people and animal disease reservoirs as the wild meat industry expanded recently. Global warming will likely contribute to the spread of dengue beyond tropical regions (Tony McMichael, National Centre for Epidemiology and Population Health, Canberra, Australia). Habitat fragmentation by deforestation may increase the contact between people and reservoir species. For example, hemorrhagic fever virus has been linked to deforestation in South America.

Containing an emerging disease depends on rapidly designing and implementing a control strategy appropriate to the epidemiology of the disease. Interdisciplinary and international collaboration occurred with unprecedented rapidity during the SARS outbreak. The network of laboratories in 17 countries organized by the World Health Organization (WHO) coordinated information sharing (David Heymann, WHO) and was instrumental in rapidly identifying the etiologic agent of SARS (*1*) and in fulfilling Koch's postulates (*2*) (Albert Osterhaus, Erasmus University, Rotterdam).

As is typical of an emerging disease, no vaccines or drugs to combat SARS existed, making quarantine, patient isolation, travel restrictions, and contact precautions the only means of limiting transmission. Mathematic models provided a framework for evaluating alternative control measures and making predictions about the course of the epidemic (*3**,**4*). Previously, similar models had guided public health policy, for example, in halting an outbreak of hoof and mouth disease in the United Kingdom in 2001 (*5**,**6*). One of the complications in setting parameters in an emerging disease model is the difficulty in estimating epidemiologic limits from the initially small sample sizes. Thus, openly sharing data and analysis of key model parameters are vital.

The model must be appropriate to the nature of the disease and the accuracy of the parameter estimates (*7*). Stochasticity inherent in transmission dynamics will be particularly pronounced when infection prevalence is low. Population heterogeneity and the network structure of human interactions will affect the spread of an emerging disease. In the 2003 SARS outbreak, healthcare workers were at particular risk (*8*) and acted as bridges carrying the infection from the hospital and causing community wide epidemics. High-risk "core groups" have been a major focus of HIV/AIDS models for years (*9*), but the movement of SARS patients into the core (i.e., the hospital) adds a further complication (*3*).

The two waves of SARS clusters in Toronto (Robert Maunder, Mount Sinai Hospital, Toronto) highlight the need for surveillance even after an outbreak appears extinguished. Management of the SARS epidemic also demonstrated that public service infrastructure, which affords the greatest chance of success (*3*), is essential to the rapid containment of an outbreak. In areas most affected, contact tracing was important (*10*). In Guangdong, police departments tracked down contacts of infected persons, who were then followed up for 10 days after exposure. Evaluating the surge capacity of public health services and hospitals is one way to assess the preparedness of a medical system.

The case-fatality rate is a key determinant of the public health impact of an emerging disease and was high for SARS at approximately 15% (*11*). The relationship between infectiousness and onset of symptoms is also important. Patient isolation has greater potential as a control strategy if the illness can be diagnosed before the person becomes infectious (Roy Anderson, Imperial College London). In contrast, persons infected with influenza virus are highly infectious before they become symptomatic.

The rapidity of pathogen turnover means that evolution in pathogen populations can occur on a time scale that is epidemiologically relevant. Indeed, SARS-CoV evolved during the course of the SARS outbreak in China (*12*). Similarly, influenza is perpetuated in the human population by the evolution of new antigenic variants every year (Robin Bush, University of California, Irvine) (*13*). Even if the transmissibility of an emerging disease is initially below the threshold necessary to sustain it in a population, the potential for the organism's evolution to higher levels may exist (*14**,**15*). Thus, one should not become complacent about diseases that are repeatedly introduced through zoonosis, but teeter on the edge of sustainability within the human population.

The success with which WHO coordinated the global collaboration in containing SARS galvanized the World Health Assembly to grant WHO greater authority to verify outbreaks, conduct investigations of outbreak severity, and evaluate the adequacy of control measures. The outcome of this new authority will depend on integrating the expertise of public health officials, medical doctors, and epidemiologists worldwide with guidance from disease transmission models. The SARS outbreak demonstrated that an epidemic in one part of the world is not just an individual nation's problem but a global problem.
